# Supplementary material for: Conditional chemoconnectomics (cCCTomics) as a strategy for efficient and conditional targeting of chemical transmission
Source: eLife. 2024 Apr 30;12:RP91927. doi: 10.7554/eLife.91927 (PMC11060718; doi:10.7554/eLife.91927)
Supplement: Figure 3—source data 3. [file elife-91927-fig3-data3.zip › Figure 3 source data3 part 2.zip!/Figure 3-Source data 3-part2/1063-1-CRISPResso_Report_az97gqtx.zip!/CRISPResso_on_1063_2/9.Alleles_frequency_table_around_sgRNA_ggatgtttccggccaccgaa.pdf]

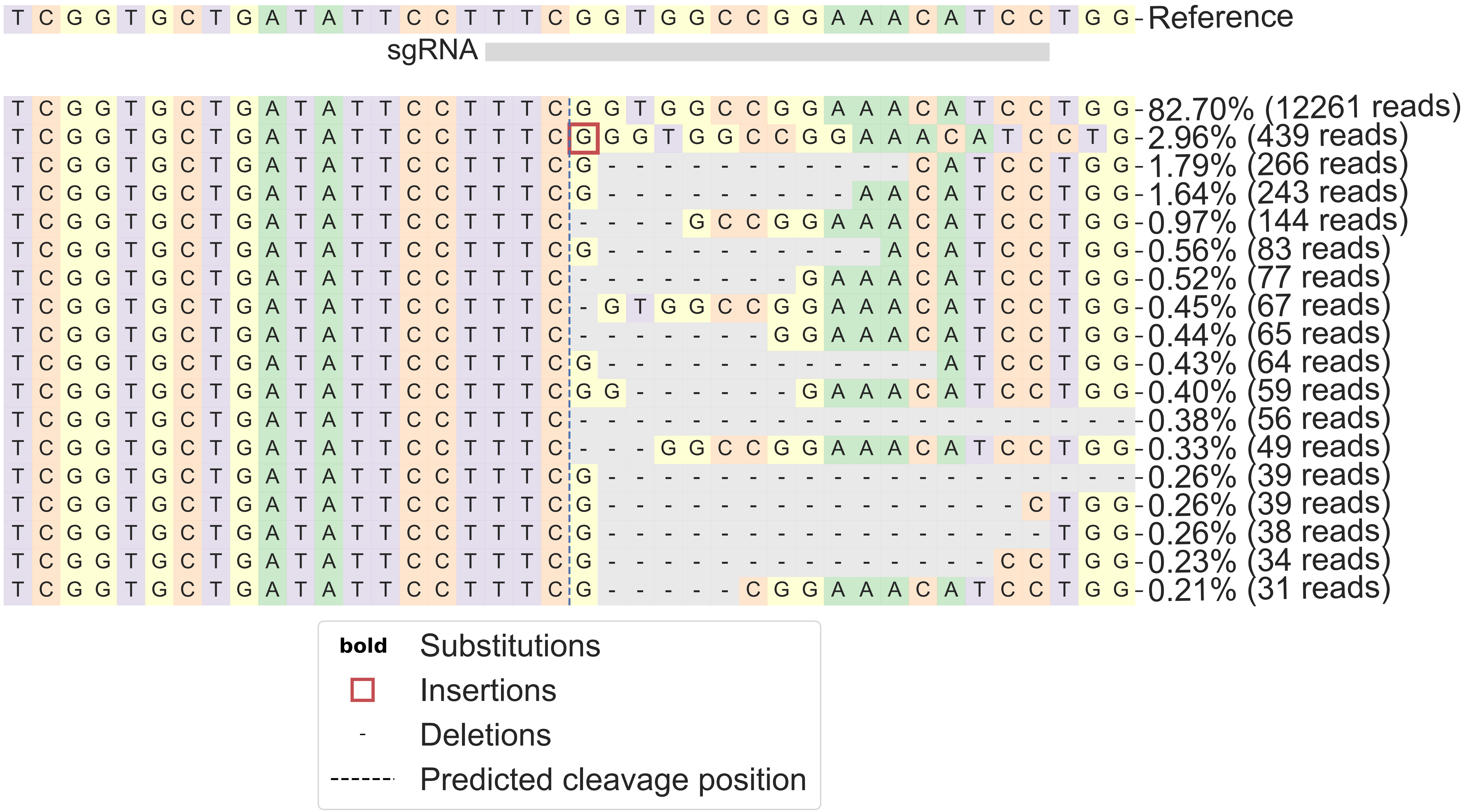

TCGGTGCTGATATTCCTTTCGGTGGCCGGAAACATCCTGG - Reference
sgRNA
82.70% (12261 reads)
2.96% (439 reads)
1.79% (266 reads)
1.64% (243 reads)
0.97% (144 reads)
0.56% (83 reads)
0.52% (77 reads)
0.45% (67 reads)
0.44% (65 reads)
0.43% (64 reads)
0.40% (59 reads)
0.38% (56 reads)
0.33% (49 reads)
0.26% (39 reads)
0.26% (39 reads)
0.26% (38 reads)
0.23% (34 reads)
0.21% (31 reads)
bold Substitutions
Insertions
Deletions
Predicted cleavage position
